# INSPIRE: Single-beam probed complementary vibrational bioimaging

**DOI:** 10.1126/sciadv.adm7687

**Published:** 2024-12-11

**Authors:** Pengcheng Fu, Yongqing Zhang, Siming Wang, Xin Ye, Yunhong Wu, Mengfei Yu, Shiyao Zhu, Hyeon Jeong Lee, Delong Zhang

**Affiliations:** ^1^ Zhejiang Key Laboratory of Micro-nano Quantum Chips and Quantum Control, School of Physics, Zhejiang University, Hangzhou 310027, China.; ^2^ Key Laboratory of Oral Biomedical Research of Zhejiang Province, Stomatology Hospital, School of Stomatology, Zhejiang University School of Medicine, Zhejiang Provincial Clinical Research Center for Oral Diseases, Cancer Center of Zhejiang University, Hangzhou 310006, China.; ^3^ Hefei National Laboratory, Hefei 230088, China.; ^4^ State Key Laboratory for Extreme Photonics and Instrumentation, Zhejiang University, Hangzhou 310027, China.; ^5^ College of Biomedical Engineering & Instrument Science, Key Laboratory for Biomedical Engineering of Ministry of Education, Zhejiang University, Hangzhou 310027, China.; ^6^ MOE Frontier Science Center for Brain Science & Brain-Machine Integration, Zhejiang University, Hangzhou 310027, China.

## Abstract

Molecular spectroscopy provides intrinsic contrast for in situ chemical imaging, linking the physiochemical properties of biomolecules to the functions of living systems. While stimulated Raman imaging has found successes in deciphering biological machinery, many vibrational modes are Raman inactive or weak, limiting the broader impact of the technique. It can potentially be mitigated by the spectral complementarity from infrared (IR) spectroscopy. However, the vastly different optical windows make it challenging to develop such a platform. Here, we introduce in situ pump-probe IR and Raman excitation (INSPIRE) microscopy, a nascent cross-modality spectroscopic imaging approach by encoding the ultrafast Raman and the IR photothermal relaxation into a single probe beam for simultaneous detection. INSPIRE inherits the merits of complementary modalities and demonstrates high-content molecular imaging of chemicals, cells, tissues, and organisms. Furthermore, INSPIRE applies to label-free and molecular tag imaging, offering possibilities for optical sensing and imaging in biomedicine and materials science.

## Teaser

INSPIRE microscopy offers complimentary chemical imaging by concurrent IR and Raman detection in cells and tissues.

## INTRODUCTION

Multimodal imaging synergistically combines multiple modalities to provide complementary insights into various targets, offering a holistic view of molecular dynamics for a comprehensive chemical, physical, and biological analysis. For example, fluorescence modality has been introduced to complement the specificity inadequacy of digital holography and interferometric scattering imaging, which are ultra-sensitive to refractive index and nanoparticle scattering, demonstrating the capability of multimodality in studying organelle interactions (*1*, *2*) and nanoscopic tracking of dynamic events (*3*, *4*). With the challenges associated with labeling small molecules and metabolites, label-free imaging by capturing intrinsic molecular structure information has been developed to complement limited molecular species (*5*). Notably, nonlinear optical processes, including second harmonic generation, transient absorption, stimulated Raman scattering (SRS), and third-order sum-frequency generation (TSFG), are systematically compatible and provide insights into the investigations of cell metabolism (*6*, *7*) and pathological mechanisms (*8*–*10*). In recent years, pump-probe sensing of absorption-induced photothermal effect (*11*, *12*) has seen an explosion with the emergence of various imaging modalities, including thermal lensing (*13*), photoacoustic detection (*14*), phase sensing (*15*–*18*), and fluorescence readout (*19*, *20*), especially for mid-infrared photothermal (MIP; also known as O-PTIR) microscopy with commercial devices available (*21*). These methods have collectively demonstrated substantial synergistic effects, leading to substantial improvements in resolution (*13*, *14*), sensitivity (*19*, *22*), and throughput (*15*, *17*) of label-free vibrational imaging.

To noninvasively probe intrinsic fingerprints of molecular vibrations, infrared (IR) and Raman spectroscopies have found fruitful applications in various fields (*23*–*26*), especially for IR with extremely high cross section (~10^−18^ cm^2^) compared with Raman at typical 10^−28^ cm^2^. However, a single modality is insufficient for assessing molecular structural information because Raman-active bands are not necessarily IR active, and vice versa (*27*), leading to partial spectral information. Therefore, it necessitates the integration of IR and Raman for complementary vibrational spectroscopic information for an in-depth understanding of complex processes (*28*–*39*). For example, the integrated IR and Raman measurement has demonstrated its capability in chemistry for identifying adsorbed NO*_x_* species (*28*) and visualizing position-dependent NO*_x_* storage efficiency (*29*) during the heterogeneous catalytic process. A recent study of CO reduction reaction has revealed the mechanism of CO subpopulation formation during adsorption with in situ surface-enhanced IR and Raman spectroscopies (*39*). Early endeavors with sequential IR and Raman imaging of biological samples have shown their potential for providing a full spectrum of cellular composition and enhancing biomarker identification (*30*, *34*).

Nonetheless, a direct combination of IR and Raman modalities is not feasible because of the distinct physical natures (absorption versus scattering) and spectral windows (mid-IR versus visible). Various endeavors have been made to combine IR and Raman for simultaneous imaging. For example, IRaman (*40*) combined MIP with a single-point Raman spectrum. To circumvent the conflicting selection rules, nonlinear optical processes were introduced, including two-photon excited Raman, i.e., hyper-Raman spectroscopy (*41*), and complementary vibrational spectroscopy by intra-pulse difference frequency generation with Fourier-transformed coherent anti-Stokes Raman scattering (FT-CARS) for single-point spectroscopic measurement (*42*). Furthermore, to improve imaging resolution, a three-photon process was adopted by combining IR-sensitive TSFG with CARS (*43*, *44*).

Here, we introduce a robust cross-modality spectroscopic imaging approach by matching the temporal signatures of optical processes with the features of the probe pulse train, termed in situ pump-probe IR and Raman excitation (INSPIRE) microscope. Specifically, each picosecond probe pulse induces stimulated Raman loss (SRL), while the probe pulse train serves as a quasi-continuous wave to detect the photothermal modulation at the microsecond timescale, with respective excitation pulses. INSPIRE imaging presents major advantages toward providing complementary spectroscopic insights of molecular structures and dynamics: (i) INSPIRE successfully overcomes the physical barriers and demonstrates simultaneous IR and Raman imaging at each pixel with submicrometer resolution, featuring ultra-sensitive photothermal detection of IR absorption and Raman amplification by stimulated emission. (ii) INSPIRE enables in situ spectroscopic complementarity, overcoming the inactive bands in Raman and IR for real-time quantitative tracking of molecular structures and dynamics. Furthermore, it is compatible with recent molecular tag imaging techniques. (iii) INSPIRE imaging is compatible and applicable to a broad range of optical processes such as two-photon excited fluorescence. Meanwhile, the detection strategy of INSPIRE can be extended to various light-matter interactions for high-content multimodal imaging. These figures of merit distinguish INSPIRE in complementary vibrational spectroscopy and imaging methods as summarized in table S1, allowing INSPIRE to bring insights into various biochemical studies, including chemical reaction monitoring, metabolic profiling during stem cell differentiation, pathology analysis, and organism imaging, as demonstrated below.

## RESULTS

### Principle and setup of INSPIRE microscope

**Fig. 1.**
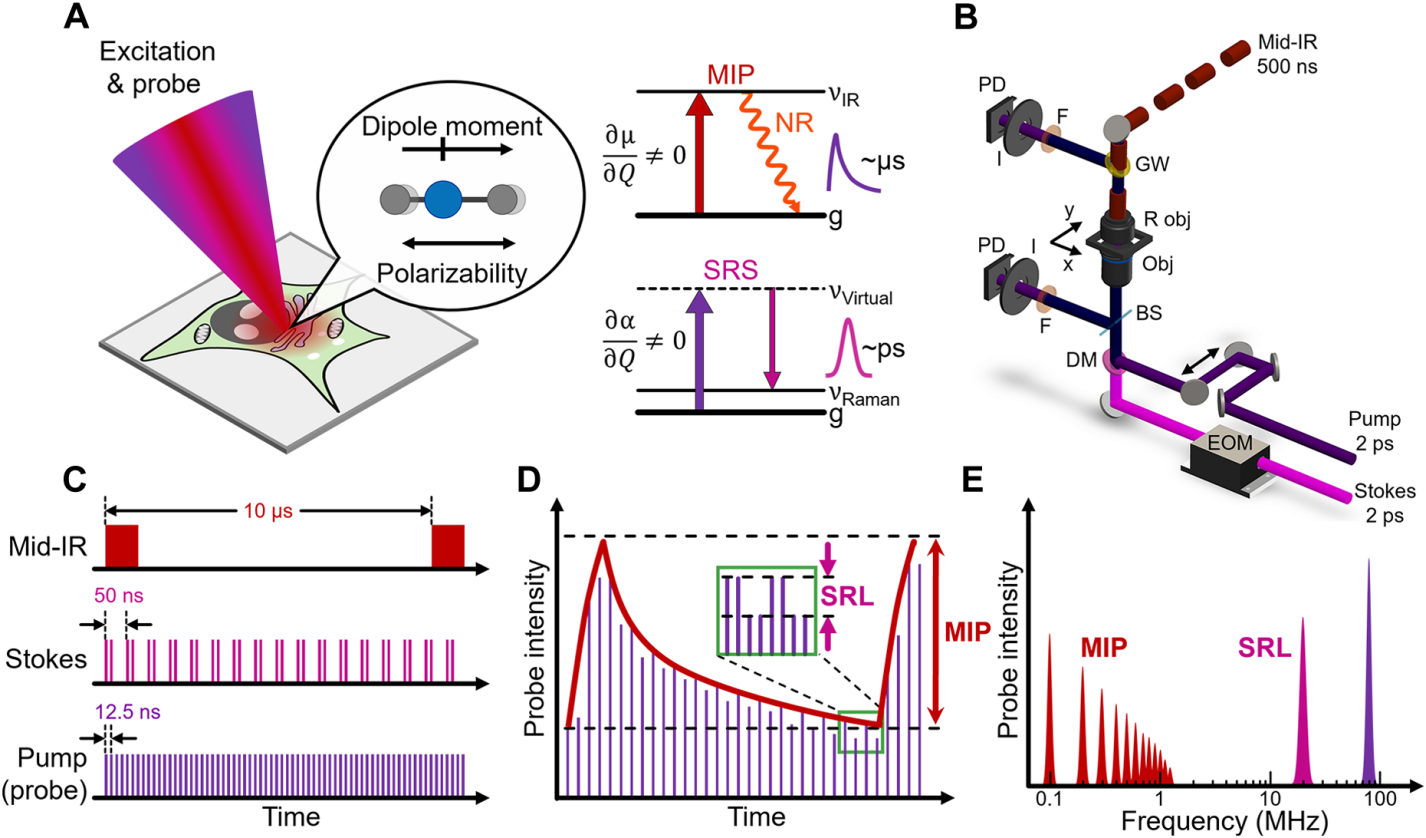
Principle and setup of INSPIRE microscopy. (**A**) INSPIRE excitation and the selection rules. α, polarizability; μ, dipole moment; *Q*, normal coordinate; NR, nonradiative relaxation; g, ground state. Note that the energy levels are not to scale. (**B**) INSPIRE microscope setup. EOM, electro-optical modulator; BS, beam splitter; DM, dichroic mirror; F, filter; I, iris; GW, germanium window; Obj, objective; PD, photodiode; R obj, reflective objective. (**C**) Pulse train for INSPIRE excitation. The mid-IR, Stokes, and pump beams were modulated at 100 kHz, 20 MHz, and 80 MHz, respectively. The dimensions are not to scale for better illustration. (**D**) Probe beam intensity after INSPIRE process, comprising MIP (red envelope) and SRL (zoomed-in view at green box) modulation. (**E**) Probe intensity shown in frequency domain.

The principle of INSPIRE microscopy is, using a single probe beam, to extract multiple light-matter interactions (Fig. 1), including the ultrafast SRS process (change in molecular polarizability ∂α/∂*Q* ≠ 0) and the relatively slow photothermal relaxation after IR absorption (change in molecular dipole moment ∂μ/∂*Q* ≠ 0). We noticed the temporal disparity between these two processes, i.e., sub-picosecond and microsecond scales after excitation pulses (Fig. 1A), which is feasible to simultaneously excite by shining the excitation pulses into the same focal volume (Fig. 1B). Notably, the detection can be achieved in a surprisingly simple but reliable way by the same probe laser, in which each single ultrafast pulse induces SRL, while the pulse train serves as a quasi-continuous wave photothermal probe (Fig. 1, C and D). For optimal photothermal excitation, we adopt a nanosecond IR pulse train (*13*). Meanwhile, for better imaging resolution, the tunable 800-nm picosecond beam was used as the probe (Fig. 1D). The signal can then be extracted at the clearly separated frequency windows by digital demodulation (Fig. 1E). Such approach solves the problems of simultaneous detection of scattering and absorption, and overcomes the optical window mismatch. It also improves molecular specificity by fully utilizing the wealth of cross-modality vibrational spectroscopy with a broader degree of freedom in spectral range.

### Performance of INSPIRE microscope

**Fig. 2.**
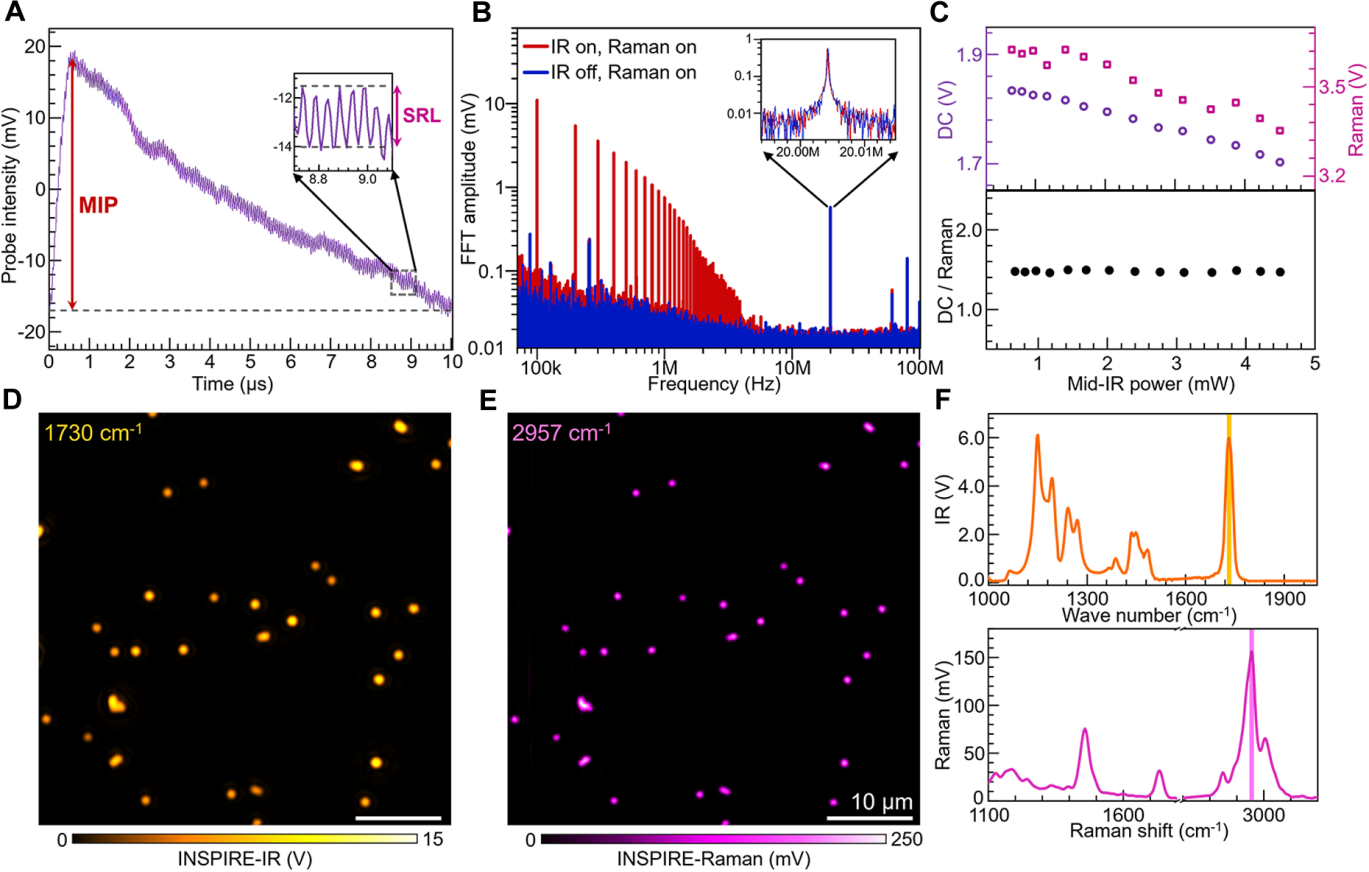
Performance of INSPIRE imaging and signal characterization. (**A**) Time domain INSPIRE signal of oil film, excited at IR 1750 cm^−1^ and Raman 2850 cm^−1^. The probe signal was ac filtered. (**B**) Power spectrum of INSPIRE signal measured at IR on (red) and off (blue) states. Insets show SRL modulation at 20 MHz. (**C**) Mid-IR power dependence of dc (total probe photons) and Raman (SRL photons), and the ratio of dc and Raman signal. (**D** and **E**) INSPIRE imaging of 500-nm PMMA beads with large field of view, excited at IR 1730 cm^−1^ C═O stretching band (D) and Raman 2957 cm^−1^ CH_3_ asymmetric stretching band (E). The output gain of lock-in amplifier for IR and Raman channels was 100 and 1000, respectively. Pixel dwell time, 12 ms. (**F**) INSPIRE spectra acquired at a single PMMA bead.

We characterized signal features by directly measuring raw signals in the temporal and frequency domains (Fig. 2, A and B), further validating the feasibility of the INSPIRE concept. The quasi-continuous probe successfully detected IR photothermal and transient SRL signals within their respective temporal windows. Notably, photothermal modulation exhibited a higher modulation depth compared to SRL modulation, attributed to larger IR cross section than SRS (Fig. 2A). Frequency-domain features of INSPIRE signals were confirmed by frequency analysis of the oil film signal (Fig. 2B), revealing major peaks at 100 kHz (with harmonics up to 5 MHz), 20 MHz, and 80 MHz, corresponding to photothermal, SRL, and probe repetition rates, respectively. No frequency mixing peaks, i.e., at 20 ± 0.1 MHz, were observed, indicating minimal cross-talk. With the IR beam on, the SRS signal shows a slight decrease (~1.8% per milliwatt average mid-IR power), which is proportional to the probe power variations from the photothermal process (Fig. 2C). Moreover, due to the large ground state population and fast dephasing of vibrational excited states at the sub-picosecond level, i.e., nondepleted pumping (*45*), the IR and Raman processes have minimal effects on each other, which is further validated experimentally (fig. S1).

To evaluate the performance of cross-modality imaging and spectroscopy, we conducted INSPIRE imaging of 500-nm poly(methyl methacrylate) (PMMA) beads at IR 1730 cm^−1^ C═O stretching band and Raman 2957 cm^−1^ CH_3_ asymmetric stretching band (Fig. 2, D and E). Three-dimensional INSPIRE imaging of a single PMMA bead (fig. S2, A and B) was performed to quantify the spatial resolution. The deconvoluted lateral resolutions of INSPIRE-Raman and INSPIRE-IR imaging were 398 and 561 nm, respectively (fig. S2C and table S2), which are well suited for subcellular cross-modality imaging. No discernible focal plane shift was observed between the INSPIRE-IR and INSPIRE-Raman imaging in the *X*-*Z* images (fig. S2, D and E), ensuring the spatial sampling accuracy for cross-modality quantitative imaging in three-dimensional specimens. To demonstrate in situ spectroscopy, simultaneous IR and Raman spectrum of a single PMMA bead was acquired by scanning the respective excitation wavelengths (Fig. 2F), demonstrating high spectral fidelity with conventional Fourier-transformed IR (FTIR) and Raman results (fig. S3).

### High-efficiency chemical imaging by INSPIRE

**Fig. 3.**
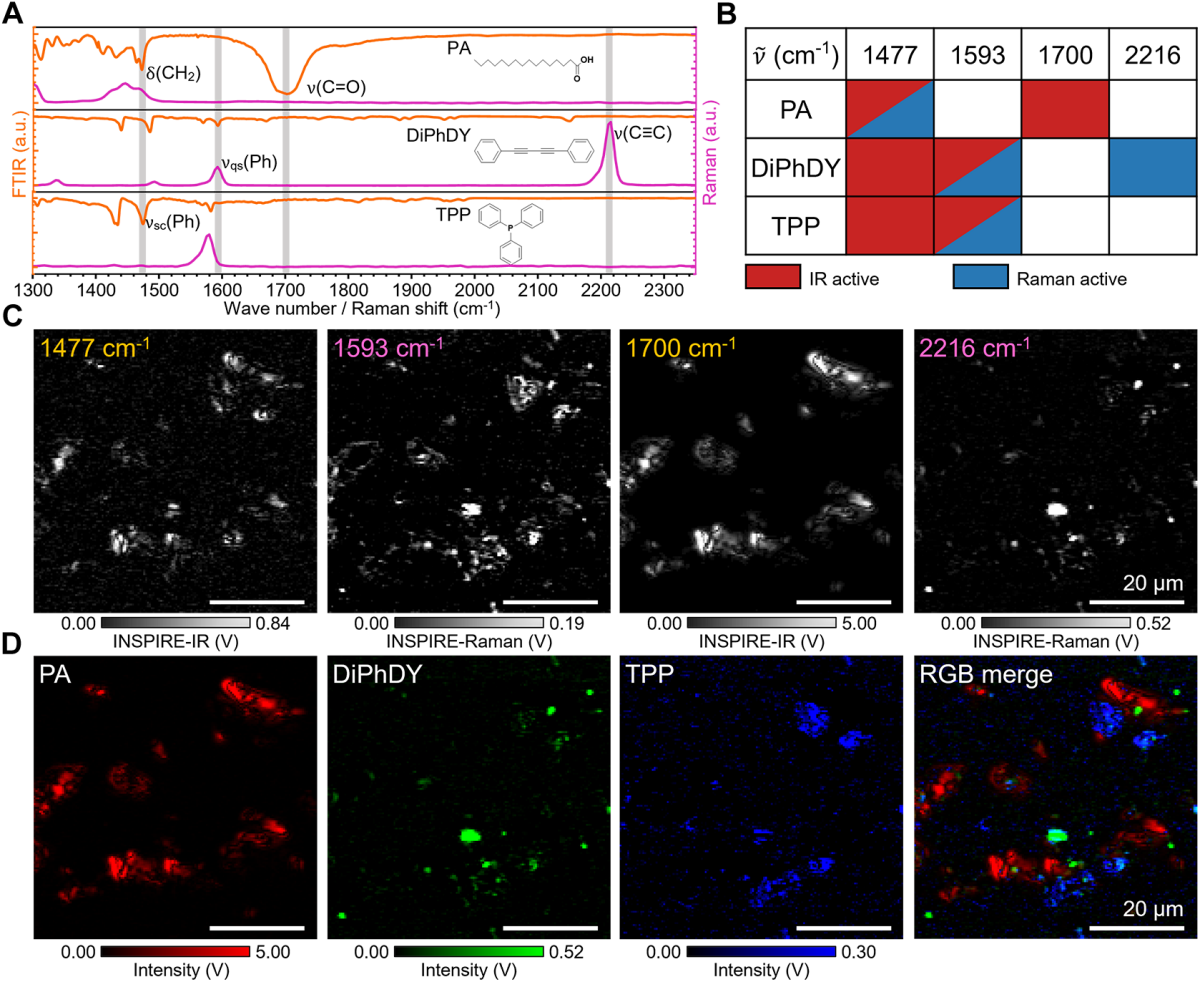
INSPIRE imaging of three chemical mixtures. (**A**) Fourier-transformed IR (FTIR) and Raman spectra of palmitic acid (PA), 1,4-diphenylbutadiyne (DiPhDY), and triphenyl phosphine (TPP). (**B**) IR and Raman activity of three chemical mixtures at selected wave numbers. (**C**) INSPIRE imaging at 1477 cm^−1^ (IR), 1593 cm^−1^ (Raman), 1700 cm^−1^ (IR), and 2216 cm^−1^ (Raman). (**D**) Pure components unmixed from INSPIRE images in (C) and the RGB composite image.

We performed INSPIRE imaging of a tertiary mixture comprising compounds relevant to biology and pharmaceuticals, including palmitic acid (PA), 1,4-diphenylbutadiyne (DiPhDY), and triphenyl phosphine (TPP) (Fig. 3). These representative molecules exhibit unique spectral peaks in both IR and Raman spectra, which can be identified using spectroscopy. However, in imaging scenarios, acquiring a whole spectrum at each pixel is challenging. Here, in the INSPIRE scheme, many peaks are exclusively found in either IR or Raman spectra (Fig. 3, A and B), reducing the chances of overlapping bands, thus mitigating the challenge associated with multispectral imaging. Even for peaks appearing in both modalities, they often differ in peak positions or spectral shapes (Fig. 3A and fig. S4, A to C). As demonstrated experimentally, INSPIRE differentiates the above three compounds in only two imaging scans, showing promise in high-efficiency chemical imaging (Fig. 3, C and D). Furthermore, INSPIRE shows better specificity compared with single-modality images at the same wave numbers by Pearson’s correlation coefficient (PCC) of the resulting images (fig. S4D; INSPIRE: 0.01, 0.08, 0.11, 0.18; IR: 0.65; Raman: 0.56).

### Metabolic profiling during stem cell differentiation with INSPIRE microscopy

**Fig. 4.**
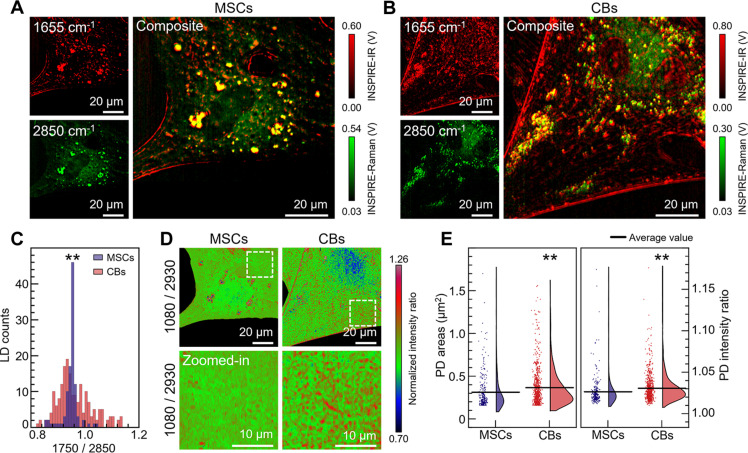
Metabolic profiling of early differentiation of MSCs to CBs with INSPIRE imaging. (**A** and **B**) INSPIRE imaging of MSCs (A) and CBs (B) performed at IR 1655 cm^−1^ amide I band and Raman 2850 cm^−1^ CH_2_ band and the composite images. (**C**) Lipid droplet (LD) distribution with relative intensity (*n* = 105 for two MSCs and *n* = 190 for two CBs). (**D**) Ratiometric images (1080 cm^−1^/2930 cm^−1^) of MSCs and CBs (top) and the zoomed-in views indicated at white dashed boxes (bottom). (**E**) Proteoglycan droplet (PD) distribution with droplet size and relative intensity of protein at Raman 2930 cm^−1^ band (*n* = 258 for two MSCs, *n* = 779 for two CBs).

Single-cell profiling is important for tracking cell differentiation process and studying its role in disease models or tissue engineering for various clinical applications. Conventional approaches for characterizing phenotypic changes are often time consuming and invasive (*46*), leading to limitations in temporal and spatial resolutions. Consequently, there is a need for techniques that facilitate noninvasive monitoring of molecular dynamics, especially during the initial phases of cellular differentiation. With the validated high-resolution and high-specificity chemical imaging capability, we demonstrate the potential of INSPIRE microscopy for in situ metabolic profiling of mesenchymal stem cells (MSCs) and differentiated chondroblasts (CBs) (Fig. 4). Specifically, INSPIRE imaging of fixed MSCs and CBs after 3-day differentiation was performed at IR 1655 cm^−1^ amide I band and Raman 2850 cm^−1^ CH_2_ skeleton vibration (Fig. 4, A and B). Leveraging the spatio-spectral information provided by INSPIRE imaging, intracellular structures, including lipid droplets (LDs), protein droplets, membranes, nuclei, and nucleoli, were clearly identified in CBs (fig. S5A). Notably, there is an increase in IR 1655 cm^−1^ intensity in the fibril-shaped membranes of CBs compared to MSCs (Fig. 4, A and B), which could be attributed to the enhanced synthesis of type II collagen during the chondrogenic differentiation process. The simultaneous Raman 2850 cm^−1^ signal revealed an increased number of LDs in CBs compared to MSCs (Fig. 4, A and B).

To further investigate the metabolic changes associated with LD synthesis, INSPIRE imaging at IR 1750 cm^−1^ C═O band and Raman 2850 cm^−1^ CH_2_ symmetric stretching band was performed, which reflect carbonyl levels (shows strong IR activity) and chain length (Raman active) in lipids, respectively (fig. S5, B and C). Statistical analysis of ratiometric intensity (1750 cm^−1^/2850 cm^−1^) revealed a significant increase in LD quantity with a broader distribution in size (Fig. 4C; *P* = 0.0070), indicating alterations in lipid synthesis following the induction of differentiation. Considering the role of LD in antioxidation (*47*), the increased LD at the early stages of chondrogenic differentiation may indicate the activation of antioxidant pathways for mitigating the accumulation of reactive oxygen species (*48*).

The synthesis of complex macromolecules, such as proteoglycans, stands as a pivotal metabolic indicator for chondrogenic differentiation. Although IR 1080 cm^−1^ has been recognized as the signature peak for proteoglycans in various IR spectroscopic studies (*49*, *50*), limitations in spatial resolution and compatibility with cellular imaging in aqueous environments in conventional IR imaging hindered the in situ mapping of proteoglycan distribution. Here, we implement ratiometric imaging at characteristic spectral bands (fig. S5D), including IR mapping at 1080 cm^−1^, representing proteoglycan (fig. S6A), and Raman mapping at 2930 cm^−1^ CH_2_ asymmetric stretching band, representing the overall protein distribution (fig. S6B). By ratiometric analysis of INSPIRE images (1080 cm^−1^/2930 cm^−1^), a substantial increase in droplet structures is observed in CBs (Fig. 4D). These droplets are most likely to be proteoglycan droplets (PDs) according to the spectral features in IR and Raman (fig. S6). Furthermore, we quantified the size and relative intensity distribution (Fig. 4E), revealing significant differences in PD synthesis (*P* = 0.0011 for size distribution, *P* = 0.0012 for intensity distribution), even at the early differentiation stage (3 days after induction). The change in PCC after differentiation (fig. S5E) further underscores the higher specificity offered by the INSPIRE microscope compared to single-modality measurements. In conclusion, these results demonstrate INSPIRE as a noninvasive, rapid single-cell profiling technique with high sensitivity, specificity, and spatial resolution without any labeling or staining.

### Identification of molecular signatures for Alzheimer’s disease by INSPIRE imaging

The identification of molecular signatures at the cellular level plays a pivotal role in the early detection and investigation of disease pathogenesis. While omics-based techniques excel at screening disease-related molecular markers, imaging-based approaches offer valuable spatial information, enabling deeper insights into molecular mechanisms. Nevertheless, conventional histopathologic analysis requires previous knowledge and labor-intensive preparations, such as immunofluorescence staining. Moreover, efficiently labeling most metabolites for in situ compositional analysis, including lipids and cholesterol, presents considerable challenges. INSPIRE addresses these challenges by offering label-free imaging with high molecular specificity, facilitating metabolic profiling in pathological conditions. The capability for in situ high-content chemical mapping and analysis is demonstrated in Alzheimer’s disease (AD) model in this study.

**Fig. 5.**
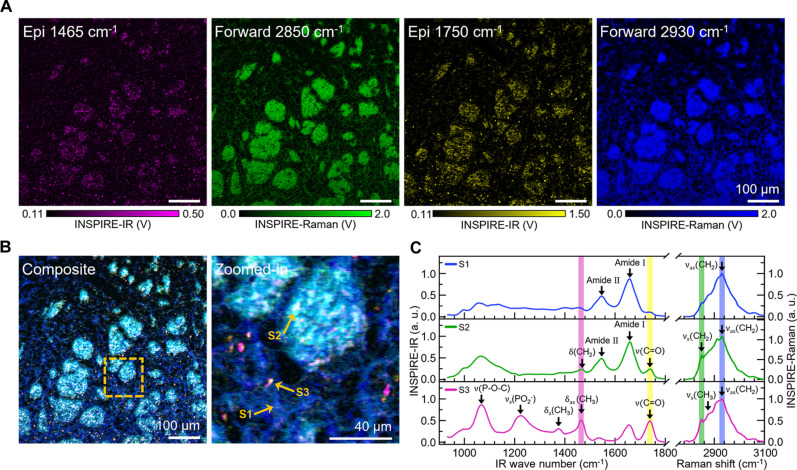
INSPIRE imaging of AD mouse striatum. (**A**) INSPIRE imaging at 1465 cm^−1^ (IR), 2850 cm^−1^ (Raman), 1750 cm^−1^ (IR), and 2930 cm^−1^ (Raman). Epi- and forward detection were used for INSPIRE-IR and INSPIRE-Raman measurement, respectively. (**B**) Composite image in (A) and zoomed-in view indicated at yellow dashed box. (**C**) INSPIRE spectra acquired at positions indicated in the yellow arrows in (B).

Benefiting from the cross-modality capabilities and the independent, broad tunability of the INSPIRE spectral region, we selected IR wave numbers at 1465 and 1750 cm^−1^ for high-quality and high-specificity fingerprint imaging of LDs, and selected Raman wave numbers at 2850 and 2930 cm^−1^ for comprehensive and reliable mapping of tissue structure in the striatum (Fig. 5A; reflection imaging of the entire brain slice is provided in fig. S7, A and B). Within the striatum, fibrous structures were uniformly distributed, prominently highlighted by the protein signal at 2930 cm^−1^. We observed the presence of striosomes ranging from 20 to 100 μm in size, characterized by high levels of both lipids and proteins (Fig. 5B). Benefiting from the distinctive sensitivity to small structure of photothermal detection scheme, INSPIRE IR channel clearly visualized a substantial number of droplets at IR 1750 cm^−1^ (representing the lipid C═O band) and IR 1465 cm^−1^ (representing CH_2_ vibrations of lipids and cholesterol-methyl), primarily located outside the striosomes with some colocalization.

To gain deeper chemical insights, in situ INSPIRE spectra were acquired from different structures, including fibrous structures (S1), droplets colocalized in the striosome (S2), and droplets outside the striosomes (S3), revealing their distinct compositions (Fig. 5, B and C). Spectra 1 and 2 prominently showed peaks at 1655 cm^−1^ (amide I), 1545 cm^−1^ (amide II), and 2930 cm^−1^ (CH_2_ asymmetric band), which are characteristics of protein components in the fibrous structure and striosomes. Notably, the presence of peaks at 1750 and 2850 cm^−1^ in spectrum 2 indicates high lipid content in striosomes compared to the fibrous structures. Furthermore, elevated cholesterol levels in the colocalized droplets were validated through multiple cholesterol characteristic peaks: IR 1375 cm^−1^ (CH_3_ symmetric bending), IR 1465 cm^−1^ (CH_3_ asymmetric bending), and Raman 2870 cm^−1^ (CH_3_ symmetric stretching). Spectrum 3 exhibits prominent peaks at 1069 and 1224 cm^−1^, associated with P─O─C and PO_2_^−^ vibrations, indicating a high phospholipid content. These results demonstrate the potential of complementary spectroscopic imaging and in situ spectroscopic measurements (fig. S7C) in identifying metabolic signatures of pathological conditions, which holds promise for facilitating disease detection and targeted therapy.

### Lipid/protein mapping in whole *Caenorhabditis elegans* by INSPIRE imaging

**Fig. 6.**
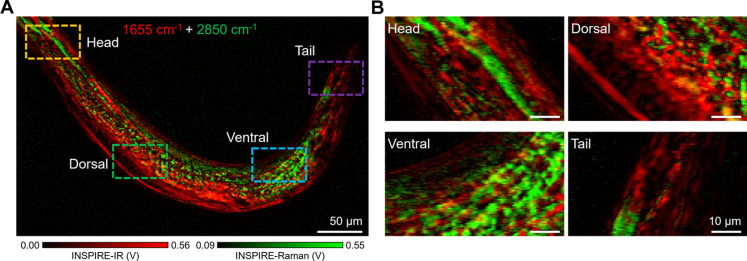
INSPIRE imaging of whole *C. elegans*. (**A**) Composite image performed at IR 1655 cm^−1^ and Raman 2850 cm^−1^. (**B**) Zoomed-in images of the head, dorsal, ventral, and tail sites, indicated at the dashed boxes in (A).

Besides single-cell profiling and metabolic imaging of tissue sections, we demonstrated that the INSPIRE microscope can be a versatile tool for investigating microorganisms. *C. elegans* is a widely adopted model organism for mechanism studies associated with aging and diseases (*51*). The different molecules mapping for mechanism studies requires at least dual wave number imaging. Simultaneous detection further enables multispectral analysis in living organisms and eliminates motion artifacts of organelles. We performed single-shot dual wave number imaging with broad tunability in anesthetized *C. elegans* at IR 1655 cm^−1^ and Raman 2850 cm^−1^, which referred to protein amide I band and lipid CH_2_ band (Fig. 6A and fig. S8, A and B). The composite image revealed the structure and biological composition of different organs inside *C. elegans* (Fig. 6B). Specifically, the lipid component was shown to store mainly in the ventral, which could be attributed to organs such as intestine. In contrast, the protein-rich domains were found to be mainly located on the dorsal, which contains rich body wall muscles. These results suggest the presence of different organ functions, demonstrating the potential of the INSPIRE microscope for studying metabolism and functions at the organism levels.

## DISCUSSION

Here, we developed an INSPIRE microscope capable of simultaneous resolution-matched IR and Raman imaging and spectroscopy with a single probe beam, offering several key advantages: (i) The IR-Raman complementarity can circumvent weak or inactive spectral bands in single-modality imaging, thereby enhancing the efficiency of chemical imaging. (ii) INSPIRE addresses the commonly known issue of low SRS signal in the fingerprint region by incorporating MIP modality that offers strong fingerprint signals due to larger absorption cross section and sensitive photothermal detection. For example, the measured SRS signal of PMMA beads at 1459 cm^−1^ is about six times lower than the signal at 2850 cm^−1^ (fig. S9). Meanwhile, this 2850 cm^−1^ SRS signal is 22 times lower in signal-to-noise ratio (SNR) than the INSPIRE-IR fingerprint signal at 1750 cm^−1^ (Fig. 2D). (iii) INSPIRE allows independent spectral tuning for each spectral channel, offering degrees of freedom such as simultaneous fingerprint and high–wave number imaging that would be otherwise difficult to realize in multiplex SRS (*52*–*54*).

Compared to the plethora of vibrational photothermal microscopes developed in recent years, INSPIRE stands out with its unique features including high-content detection and higher throughput, as summarized in table S3. By using a generalized versatile pump-probe detection scheme, the INSPIRE microscope achieves simultaneous detection of absorption and scattering. Additionally, the INSPIRE concept can be extended to various spectroscopic modalities for high-content multimodal imaging, such as integration of photothermal, photoacoustic, transient absorption modalities.

The influence of mid-IR to SRL channel is an essential factor to discuss. Despite a 1.8% intensity drop from mid-IR photothermal effect, mid-IR may also affect SRL transition aspects: competition from other nonlinear effects, transient thermal population, and thermal-modulated Raman cross section on temperature-sensitive Raman bonds. The nonlinear interactions of the mid-IR beam with the near-IR beam can be excited in an ultrafast system or a cavity-enhanced system, such as bond-selective fluorescence-detected IR-excited effect (*55*) or vibrationally assisted luminescence (*56*), where conditions are not satisfied in the INSPIRE system. Thermal population–induced perturbation can be neglected, given the high Raman energy (2900 cm^−1^) and weak temperature rise, typically below 10 K, in the photothermal modulation (*57*). For temperature-sensitive bonds, especially those related to hydrogen bonds (*58*), photothermal effect may have a transient modulation on SRL intensity.

The performance of the current INSPIRE system still has space to improve. The current spectral acquisition is quasi-simultaneous due to the tuning speed mismatch between the quantum cascade laser (QCL) and optical parametric oscillator (OPO), which can be compensated with mature techniques such as spectral focusing (*59*). The imaging throughput of INSPIRE is currently constrained by the scanning stage due to sample scanning, as regular optical components are not IR transmissive. Higher imaging throughput can be achieved by implementing widefield imaging (*15*, *60*), mirror-based IR laser scanning (*61*), and parallel detection strategies. The SNR can be further improved using lock-in-free techniques (*57*, *62*), balanced detection (*63*, *64*), or probing with a quantum-enhanced laser (*65*). Furthermore, imaging resolution can be improved by probing with a shorter wavelength (*14*, *66*) and localization with harmonic concept (*67*) or algorithms (*68*). Detection efficiency can be further improved by a high-NA (numerical aperture) aspherical lens or specially designed IR objective, considering the center obscuration of the reflective objective. INSPIRE shows promise in single-molecule sensitivity with assistance of plasmon resonance (*69*, *70*). With the currently equipped picosecond-laser source, INSPIRE can be a versatile imaging and analytical platform compatible with abundant nonlinear optical imaging modalities, opening up possibilities for optical imaging and sensing for biomedicine and materials science.

## MATERIALS AND METHODS

### INSPIRE microscope

The INSPIRE microscope was constructed on an upright microscope frame (BX51, Olympus) with counter-propagation scheme (Fig. 1B). A near-IR beam (700 to 990 nm) and a 1031-nm beam with 2-ps duration and 80-MHz repetition rate were provided by a picosecond OPO laser (picoEmerald, Applied Physics & Electronics). Then, the 1031-nm beam, modulated at 20 MHz with an EOM (EO-AM-NR-C2, Thorlabs), served as the Stokes beam, while the near-IR beam served as the pump beam for the stimulated Raman transition. Two near-IR beams were colinearly combined, temporally overlapped, and focused with a 1.2-NA water-immersion objective (UPlanSApo 60×/1.20 W, Olympus). A nanosecond mid-IR beam from QCL (915 to 2335 cm^−1^, 500-ns duration, Daylight Solutions) was expanded and focused with a reflective objective (Thorlabs, LMM40X-P01) in the counter scheme. The near-IR beam with a shorter wavelength served as the probe beam, collected with the reflective objective (forward detection) and sent to a high-bandwidth homebuilt photodiode with an IR dichroic mirror (WG91050-G, Thorlabs). The 1031-nm beam was filtered with appropriate filter sets. A variable iris was set to optimize the photothermal effect as a trade-off for SRS. The voltage signal was first amplified by a low noise amplifier (NF230, NF Cooperation) and sent to a multi-channel lock-in amplifier (HF2LI, Zurich instruments) for digital demodulation of both IR and Raman signals at 100 kHz and 20 MHz, respectively. Sample scanning was performed on a three-axis piezo stage (P-562.3, Physik Instrumente) and synchronized with a data acquisition card (6361, National Instruments), controlled by a homebuilt code. The co-propagation INSPIRE microscope was customized on a commercial MIP imaging setup (mIRage, Photothermal Spectroscopy Corp.) by replacing the probe beam with picosecond beams by an IR dichroic mirror (WG91050-G, Thorlabs). Data related to Figs. 2 to 4 were acquired on the counter-propagation INSPIRE microscope. Data related to Figs. 5 and 6 were acquired on the customized co-propagation INSPIRE microscope.

### INSPIRE spectra acquisition

INSPIRE spectra were acquired with the following quasi-simultaneous steps: First, the MIP spectrum was acquired by fast tuning of QCL (100 cm^−1^/s, 2 cm^−1^ resolution) with the probe beam wavelength fixed at 780 nm. Second, the SRS spectrum was acquired by tuning the SRS pump beam wavelength (signal beam of near-IR OPO laser) with a speed of 1 cm^−1^/s and a spectral resolution of 8 cm^−1^. The INSPIRE spectrum in Fig. 2 was acquired on the counter-propagation INSPIRE microscope. INSPIRE spectra in Fig. 5 were acquired on the customized co-propagation INSPIRE microscope.

### FTIR and Raman spectra acquisition

FTIR spectra and spontaneous Raman spectra of PA (Aladdin), DiPhDY (Bidepharm), and TPP (Aladdin) powder (Fig. 3A and fig. S4, A to C) were measured separately. Raman spectra were acquired with a homebuilt Raman spectrometer (Shamrock SR-500, Andor Technology), excited with 100-mW continuous-wave 638-nm laser (LuxX 638-200, Omicron Laserage) for 30 s by a water-immersion objective (UPlanSApo 60×/1.20 W, Olympus). FTIR spectra were acquired on a commercial FTIR spectrometer (Nicolet iS10, Thermo Fisher Scientific) with 4 cm^−1^ spectral resolution, averaged for 16 times. Because of the spatial resolution of standard FTIR spectrometer, the PMMA film was used for measurements (fig. S3A). The SRS spectra are normalized to the corresponding Raman spectra (figs. S3B and S4, A to C).

### Chemical mixture imaging

PA, DiPhDY, and TPP powder were mixed with a molar ratio of 1:1:1 on a 0.5-mm CaF_2_ window and sealed with a 170-μm cover glass. INSPIRE images were taken with a pixel size of 0.4 μm and a pixel dwell time of 3 ms with 1000 and 10,000 lock-in amplifier gain for INSPIRE-IR and INSPIRE-Raman, respectively. The power of probe, Stokes, and mid-IR beams was 140, 140, and 5 mW, respectively.

### Cell imaging

Adipose-derived stem cells (ADSCs) were extracted from Sprague-Dawley rats (Ethics Committee of Animal Care and Use Committee for Teaching and Research at Zhejiang University, no. ZJU20200004). Chondrogenic differentiation was induced by medium (Cyagen) for differentiating ADSCs (MSCs) to CB cells, cultured on 0.5-mm CaF_2_ substrates. After 3 days of differentiation, CB and MSC cells were fixed with 10% formalin for 15 min and then washed and sealed with phosphate-buffered saline (PBS) by 170-μm cover glass for imaging. Cell imaging was performed with a pixel size of 0.3 μm and a pixel dwell time of 5 ms. The power of probe, Stokes, and mid-IR beams was 140, 140, and 5 mW, respectively. Gains were set at 1000 and 10,000 for INSPIRE-IR and INSPIRE-Raman channels, respectively.

### Power spectrum measurement and imaging

Oil film was sandwiched between a 170-μm cover glass and a 0.5-mm CaF_2_ window. The power of pump (at 796.8 nm), Stokes, and mid-IR (1750 cm^−1^) beam was 315, 315, and 5 mW, respectively. INSPIRE focuses were adjusted to the surface of CaF_2_ due to the limited penetration depth of mid-IR beam. Probe beam intensity was forward detected, and the signal was amplified and then recorded with a high-speed oscilloscope (DS77024, RIGOL, 10GSa/s) for acquisition (1024 times averaged, 1 ms length). Oil film imaging (1 μm pixel size and 2 ms dwell time) was performed by demodulation with a lock-in amplifier (1000 and 10,000 gains for INSPIRE-IR and INSPIRE-Raman channel, respectively).

### Brain slice imaging

The 10-μm cryosectioned fixed brain slice of 5xFAD adult mouse (9 months) was provided by the Piatkevich laboratory in Westlake University (Laboratory Animal Resources Center of Westlake University, no. 20210621KP001). INSPIRE imaging was performed on the co-propagation INSPIRE microscope with a pixel size of 0.8 μm and a dwell time of 3 ms. The power of probe, Stokes, and mid-IR beams was set to 100, 150, and 5 mW, respectively.

### *C. elegans* imaging

Wild-type (N_2_) *C. elegans* strain was provided by the W. Zou laboratory in Zhejiang University (Institute of Translational Medicine of Zhejiang University). After anesthetizing the *C. elegans*, the worm was placed on a 170-μm cover glass, sandwiched with a 0.5-mm CaF_2_ window for imaging. INSPIRE imaging (0.5 μm pixel size and 1 ms dwell time) was performed on the co-propagation INSPIRE microscope, with probe, Stokes, and mid-IR power set at 100, 150, and 5 mW, respectively.

### Image analysis

INSPIRE-IR images were first power normalized for further analysis. Spectral unmixing methods were performed in Matlab with a homebuilt code. For ratiometric analysis, each image was first subtracted with background and normalized, and then added with a constant (with value = 1) to avoid abnormal values in divisions near zero. Next, two images were divided to obtain the ratiometric image. All image processes were performed in ImageJ.

### Statistical analysis

Images were bilinearly interpolated to 0.1-μm pixel size. Droplets with circularity between 0.8 and 1.0 are extracted with minimal size threshold determined by the resolution limit of the INSPIRE system, and then the size and intensity value were measured. For statistical analysis, two-sample *t* test and two-sample variance test were used to determine statistical significance (*P < 0.05, **P < 0.01, ***P < 1 × 10^−3^, ****P < 1 × 10^−4^).

## Supplementary Materials

### This PDF file includes:

Figs. S1 to S9 Tables S1 to S3 References
