# Supplementary material for: INSPIRE: Single-beam probed complementary vibrational bioimaging
Source: Sci Adv. 2024 Dec 11;10(50):eadm7687. doi: 10.1126/sciadv.adm7687 (PMC11633736; doi:10.1126/sciadv.adm7687)
Supplement: Supplementary file 1 — Figs. S1 to S9 Tables S1 to S3 References [file sciadv.adm7687_sm.pdf]

Science Advances
AAAS

Supplementary Materials for

INSPIRE: Single-beam probed complementary vibrational bioimaging

Pengcheng Fu *et al.*

Corresponding author: Mengfei Yu, yumengfei@zju.edu.cn; Hyeon Jeong Lee, hjlee@zju.edu.cn; Delong Zhang, dlzhang@zju.edu.cn

Sci. Adv. **10**, eadm7687 (2024)
DOI: 10.1126/sciadv.adm7687

This PDF file includes:

Figs. S1 to S9
Tables S1 to S3
References

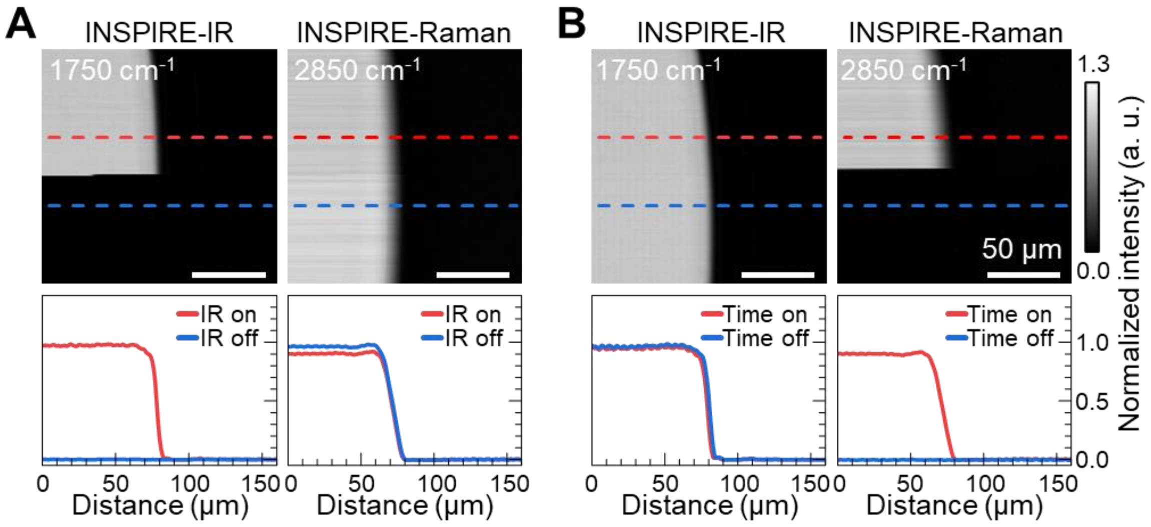

Fig. S1. INSPIRE images of oil film measured at IR off and time off states.

(A-B) Oil/air interface images taken at IR off **(A)** and time off **(B)** during scanning. The IR band was selected at 1750 cm^{-1}, and the Raman band was selected at 2850 cm^{-1}. Line profiles measured from corresponding dashed lines in **(A)** and **(B)**.

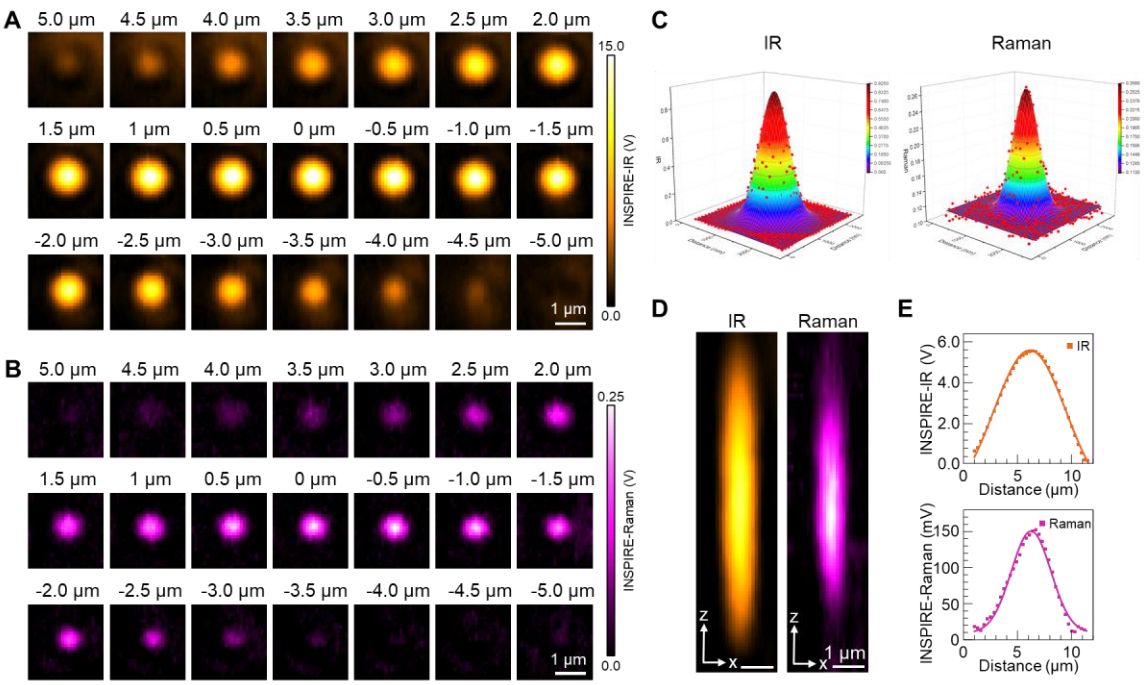

Fig. S2. Three-dimensional INSPIRE images of a 500-nm PMMA bead.

(A-B) Three-dimensional images of a 500-nm PMMA bead performed at IR 1730 cm^{-1} **(A)** and Raman 2957 cm^{-1} **(B)**. **(C)** 2D Gaussian fitting of bead in IR and Raman channels. **(D)** X-Z views of bead imaging in IR and Raman channels. **(E)** Z profiles of bead in IR and Raman channels.

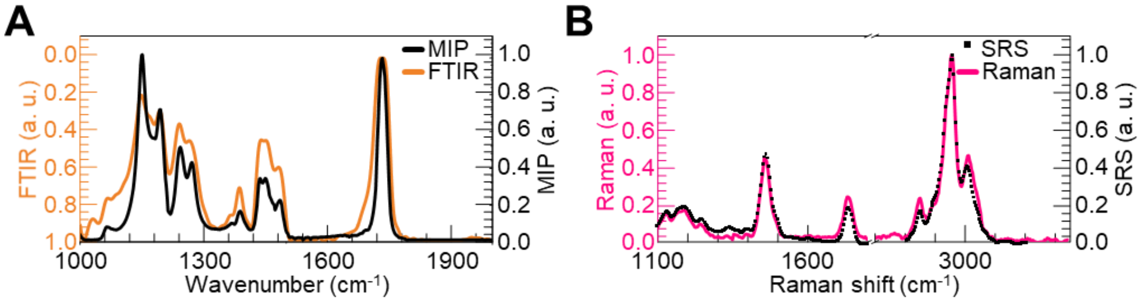

Fig. S3. Comparison of INSPIRE spectra with FTIR/Raman spectra of PMMA.
(A-B) Comparison of MIP with FTIR spectra **(A)** and SRS with Raman spectra **(B)**.

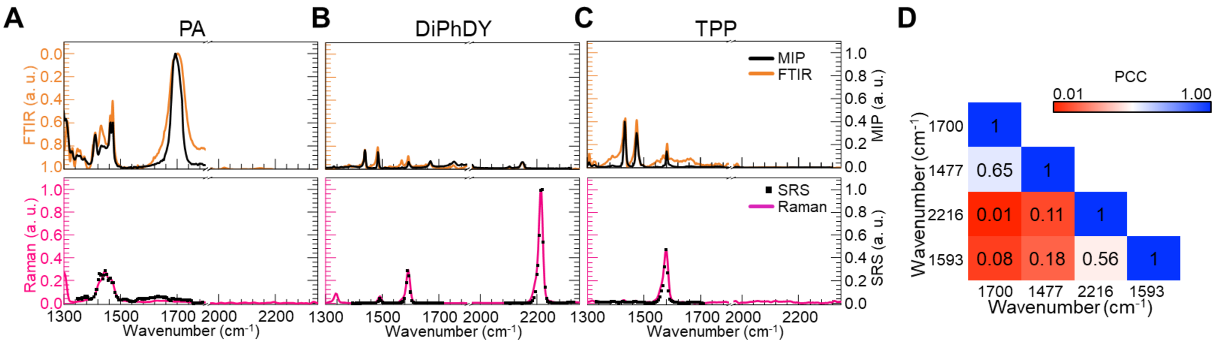

Fig. S4. High specificity of INSPIRE imaging by PCC evaluation.

(A-C) Comparison of INSPIRE spectra with FTIR/Raman spectra of PA **(A)**, DiPhDY **(B)**, and TPP **(C)**. **(D)** PCC of INSPIRE images in Fig. 3.

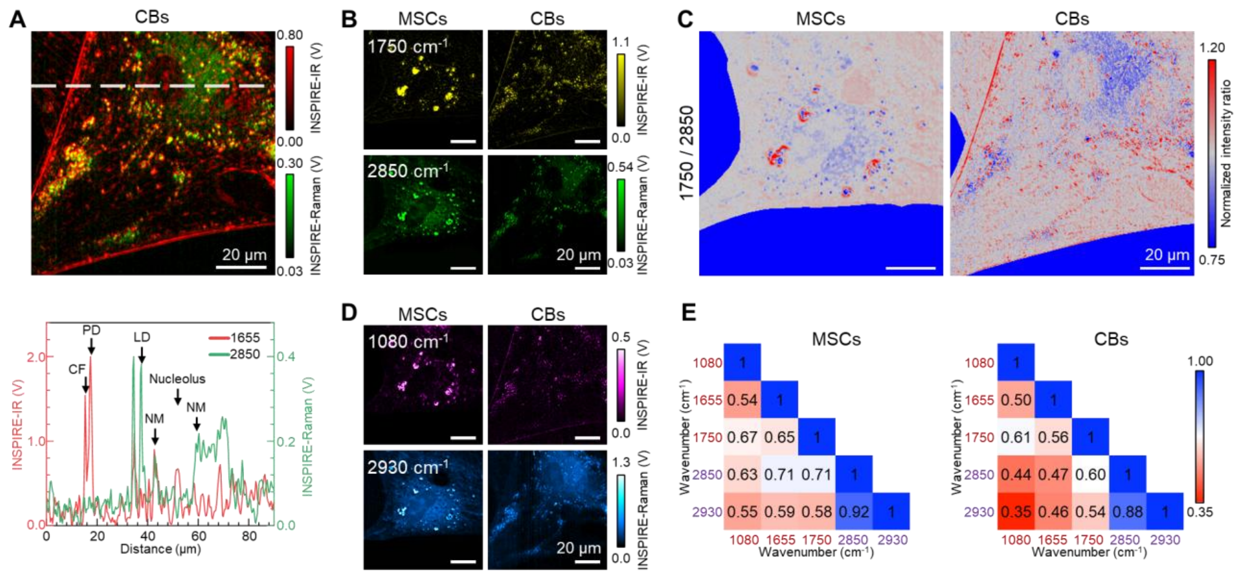

Fig. S5. INSPIRE imaging of MSCs and CBs.

(A) Composite image of CBs performed at IR 1655 cm^{-1} and Raman 2850 cm^{-1}. Line profiles indicated at white dashed line. CF, collagen fibers. PD, protein droplet. LD, lipid droplet. NM, nuclear membrane. **(B)** INSPIRE images of MSCs and CBs performed at IR 1750 cm^{-1} and Raman 2850 cm^{-1}. **(C)** Ratiometric images 1750 cm^{-1} / 2850 cm^{-1} of MSCs and CBs. **(D)** INSPIRE images of MSCs (left) and CBs (right) performed at IR 1080 cm^{-1} and Raman 2930 cm^{-1}. **(E)** PCC of MSCs and CBs images.

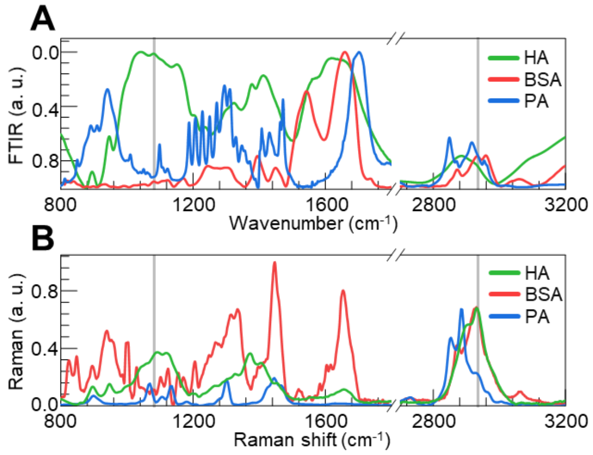

Fig. S6. FTIR/Raman spectra of three biomolecules.

(A-B) FTIR **(A)** and Raman **(B)** spectra of hyaluronic acid (HA), bovine serum albumin (BSA), and palmitic acid (PA). The shaded area shows the peak of HA at 1080 cm^{-1} and the peak of BSA at 2930 cm^{-1}.

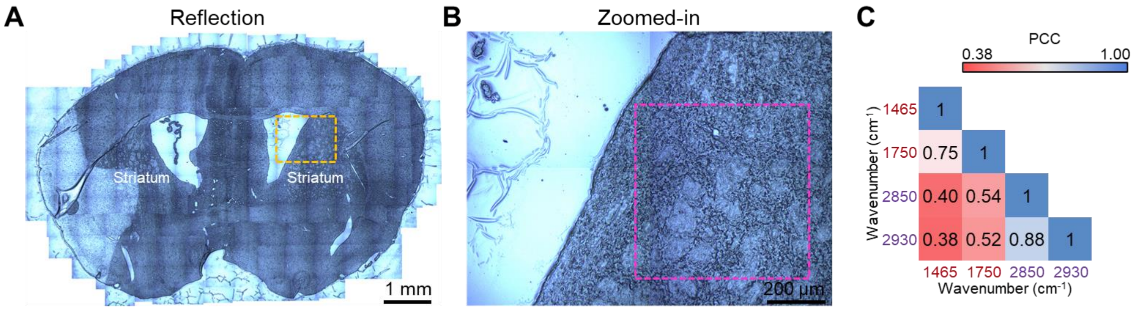

Fig. S7. Brightfield imaging of whole AD brain and region of interests for INSPIRE imaging. **(A)** Reflection image of whole AD mouse brain. **(B)** Zoomed-in view of stiratum in mouse brain indicated by yellow dashed box in **(A)**. INSPIRE imaging in Fig. 5 was performed at region of interest indicated at the magenta dashed box. **(C)** PCC of INSPIRE images.

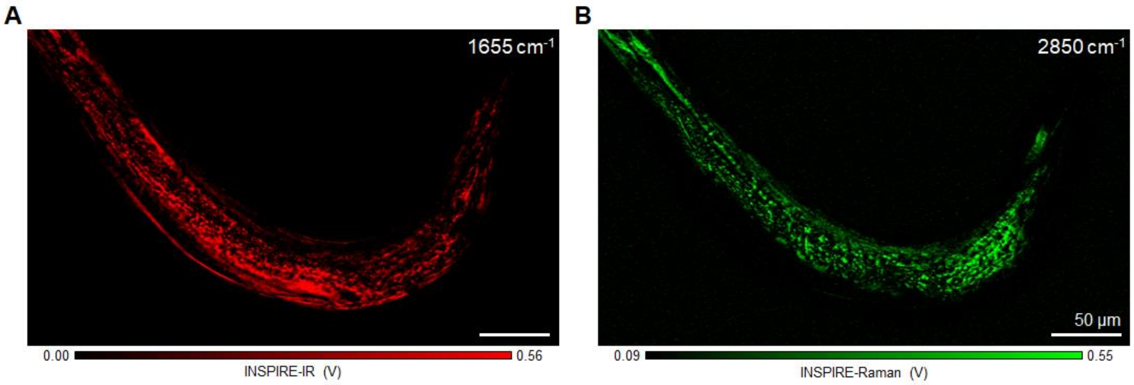

Fig. S8. INSPIRE images of whole *C. elegans*.

(A-B) INSPIRE images performed at IR 1655 cm^{-1} **(A)** and Raman 2850 cm^{-1} **(B)**.

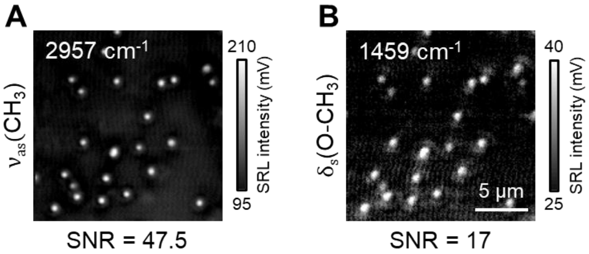

Fig. S9. SNR comparison of regular SRS microscope at high wavenumber region and fingerprint region.

(A) SRS imaging of 500-nm PMMA beads at 2957 cm^{-1}. (**B**) SRS imaging of 500-nm PMMA beads at 1459 cm^{-1}.

Table S1. Performance comparison of complementary spectroscopy and imaging methods.

Ref	Methods	Complementary imaging	Complementary spectroscopy	Simultaneous	Resolution matched	Single detector
(*28*)	FTIR + Raman spectrometer	\	+	√	×	×
(*30*)	Synchrotron FTIR + Raman	+	+	×	×	×
(*31*)	Synchrotron FTIR + CARS	+	+	×	×	×
(*36*)	AFMIR + TERS	++	+++	×	√	×
(*39*)	SEIRS + SERS	-	+++	√	×	×
(*71*)	IR photothermal + single point Raman	-	+	√	√	×
(*41*)	HyperRaman + Raman	+	++	√	√	√
(*42*)	ITSFG + FT-CARS	\	+++	√	×	×
(*43*)	TSFG + CARS	+	++	√	√	×
INSPIRE	IR photothermal + SRS	+++++	+++	√	√	√

Table S2. 2D Gaussian fitting and deconvolution results.

Modality	Raman		IR	
**Direction**	**x**	**y**	**x**	**y**
Measured FWHM (nm)	651	627	750	754
Deconvoluted FWHM (nm)	417	378	559	564
Averaged FWHM (nm)	397.6		561.7	

Table S3. Advantages of different vibrational photothermal microscopies.

Name	Super-resolved IR imaging	Raman imaging	Fluorescence imaging	Raman spectrum	Cross-modality
MIRage-LS (*21*)	√		√	single point	
FMIP (*19, 20*)	√		√		
Phase microscopy (*15-18*)	√				
SRP (*22*)		√	compatible		
INSPIRE	√	√	compatible		√

REFERENCES AND NOTES

1. D. Dong, X. Huang, L. Li, H. Mao, Y. Mo, G. Zhang, Z. Zhang, J. Shen, W. Liu, Z. Wu, G. Liu, Y. Liu, H. Yang, Q. Gong, K. Shi, L. Chen, Super-resolution fluorescence-assisted diffraction computational tomography reveals the three-dimensional landscape of the cellular organelle interactome. *Light Sci. Appl.* **9**, 11 (2020).

2. M. A. Neginskaya, S. E. Morris, E. V. Pavlov, Refractive index imaging reveals that elimination of the ATP synthase C subunit does not prevent the adenine nucleotide translocase-dependent mitochondrial permeability transition. *Cells* **12**, 1950 (2023).

3. P. Kukura, H. Ewers, C. Muller, A. Renn, A. Helenius, V. Sandoghdar, High-speed nanoscopic tracking of the position and orientation of a single virus. *Nat. Methods* **6**, 923–927 (2009).

4. J. S. Park, I. B. Lee, H. M. Moon, J. S. Ryu, S. Y. Kong, S. C. Hong, M. Cho, Fluorescence-combined interferometric scattering imaging reveals nanoscale dynamic events of single nascent adhesions in living cells. *J. Phys. Chem. Lett.* **11**, 10233–10241 (2020).

5. V. Marx, It's free imaging—Label-free, that is. *Nat. Methods* **16**, 1209–1212 (2019).

6. V. Raghunathan, Y. Han, O. Korth, N. H. Ge, E. O. Potma, Rapid vibrational imaging with sum frequency generation microscopy. *Opt. Lett.* **36**, 3891–3893 (2011).

7. H. J. Lee, Z. Chen, M. Collard, F. Chen, J. G. Chen, M. Wu, R. M. Alani, J. X. Cheng, Multimodal metabolic imaging reveals pigment reduction and lipid accumulation in metastatic melanoma. *BME Front.* **2021**, 9860123 (2021).

8. H. Tu, Y. Liu, D. Turchinovich, M. Marjanovic, J. Lyngso, J. Laegsgaard, E. J. Chaney, Y. Zhao, S. You, W. L. Wilson, B. Xu, M. Dantus, S. A. Boppart, Stain-free histopathology by programmable supercontinuum pulses. *Nat. Photonics* **10**, 534–540 (2016).

9. Y. Li, B. Shen, G. Zou, R. Hu, Y. Pan, J. Qu, L. Liu, Super-multiplex nonlinear optical imaging unscrambles the statistical complexity of cancer subtypes and tumor microenvironment. *Adv. Sci.* **9**, e2104379 (2022).

10. J. Ferrer Ortas, P. Mahou, S. Escot, C. Stringari, N. B. David, L. Bally-Cuif, N. Dray, M. Negrerie, W. Supatto, E. Beaurepaire, Label-free imaging of red blood cells and oxygenation with color third-order sum-frequency generation microscopy. *Light Sci. Appl.* **12**, 29 (2023).

11. D. Boyer, P. Tamarat, A. Maali, B. Lounis, M. Orrit, Photothermal imaging of nanometer-sized metal particles among scatterers. *Science* **297**, 1160–1163 (2002).

12. Y. Bai, J. Yin, J. X. Cheng, Bond-selective imaging by optically sensing the mid-infrared photothermal effect. *Sci. Adv.* **7**, eabg1559 (2021).

13. D. Zhang, C. Li, C. Zhang, M. N. Slipchenko, G. Eakins, J. X. Cheng, Depth-resolved mid-infrared photothermal imaging of living cells and organisms with submicrometer spatial resolution. *Sci. Adv.* **2**, e1600521 (2016).

14. J. Shi, T. T. W. Wong, Y. He, L. Li, R. Zhang, C. S. Yung, J. Hwang, K. Maslov, L. V. Wang, High-resolution, high-contrast mid-infrared imaging of fresh biological samples with ultraviolet-localized photoacoustic microscopy. *Nat. Photonics* **13**, 609–615 (2019).

15. D. Zhang, L. Lan, Y. Bai, H. Majeed, M. E. Kandel, G. Popescu, J. X. Cheng, Bond-selective transient phase imaging via sensing of the infrared photothermal effect. *Light Sci. Appl.* **8**, 116 (2019).

16. M. Tamamitsu, K. Toda, H. Shimada, T. Honda, M. Takarada, K. Okabe, Y. Nagashima, R. Horisaki, T. Ideguchi, Label-free biochemical quantitative phase imaging with mid-infrared photothermal effect. *Optica* **7**, 359–366 (2020).

17. G. Ishigane, K. Toda, M. Tamamitsu, H. Shimada, V. R. Badarla, T. Ideguchi, Label-free mid-infrared photothermal live-cell imaging beyond video rate. *Light Sci. Appl.* **12**, 174 (2023).

18. T. Yuan, L. Riobo, F. Gasparin, V. Ntziachristos, M. A. Pleitez, Phase-shifting optothermal microscopy enables live-cell mid-infrared hyperspectral imaging of large cell populations at high confluency. *Sci. Adv.* **10**, eadj7944 (2024).

19. Y. Zhang, H. Zong, C. Zong, Y. Tan, M. Zhang, Y. Zhan, J. X. Cheng, Fluorescence-detected mid-infrared photothermal microscopy. *J. Am. Chem. Soc.* **143**, 11490–11499 (2021).

20. M. Li, A. Razumtcev, R. Yang, Y. Liu, J. Rong, A. C. Geiger, R. Blanchard, C. Pfluegl, L. S. Taylor, G. J. Simpson, Fluorescence-detected mid-infrared photothermal microscopy. *J. Am. Chem. Soc.* **143**, 10809–10815 (2021).

21. Y. Bai, C. M. Camargo, S. M. K. Glasauer, R. Gifford, X. Tian, A. P. Longhini, K. S. Kosik, Single-cell mapping of lipid metabolites using an infrared probe in human-derived model systems. *Nat. Commun.* **15**, 350 (2024).

22. Y. Zhu, X. Ge, H. Ni, J. Yin, H. Lin, L. Wang, Y. Tan, C. V. Prabhu Dessai, Y. Li, X. Teng, J.-X. Cheng, Stimulated Raman photothermal microscopy toward ultrasensitive chemical imaging. *Sci. Adv.* **9**, eadi2181 (2023).

23. F. Zaera, New advances in the use of infrared absorption spectroscopy for the characterization of heterogeneous catalytic reactions. *Chem. Soc. Rev.* **43**, 7624–7663 (2014).

24. T. P. Wrobel, R. Bhargava, Infrared spectroscopic imaging advances as an analytical technology for biomedical sciences. *Anal. Chem.* **90**, 1444–1463 (2018).

25. N. Kuhar, S. Sil, T. Verma, S. Umapathy, Challenges in application of Raman spectroscopy to biology and materials. *RSC Adv.* **8**, 25888–25908 (2018).

26. Y. Shen, F. Hu, W. Min, Raman imaging of small biomolecules. *Annu. Rev. Biophys.* **48**, 347–369 (2019).

27. P. Larkin, *Infrared and Raman Spectroscopy: Principles and Spectral Interpretation* (Elsevier, 2017).

28. G. Le Bourdon, F. Adar, M. Moreau, S. Morel, J. Reffner, A. S. Mamede, C. Dujardin, E. Payen, In situ characterization by Raman and IR vibrational spectroscopies on a single instrument: DeNOxreaction over a Pd/γ-Al2O3catalyst. *Phys. Chem. Chem. Phys.* **5**, 4441–4444 (2003).

29. A. Urakawa, N. Maeda, A. Baiker, Space- and time-resolved combined DRIFT and Raman spectroscopy: Monitoring dynamic surface and bulk processes during NO_x storage reduction. *Angew. Chem. Int. Ed.* **47**, 9256–9259 (2008).

30. B. R. Wood, T. Chernenko, C. Matthaus, M. Diem, C. Chong, U. Bernhard, C. Jene, A. A. Brandli, D. McNaughton, M. J. Tobin, A. Trounson, O. Lacham-Kaplan, Shedding new light on the molecular architecture of oocytes using a combination of synchrotron Fourier transform-infrared and Raman spectroscopic mapping. *Anal. Chem.* **80**, 9065–9072 (2008).

31. M. H. Kox, K. F. Domke, J. P. Day, G. Rago, E. Stavitski, M. Bonn, B. M. Weckhuysen, Label-free chemical imaging of catalytic solids by coherent anti-Stokes Raman scattering and synchrotron-based infrared microscopy. *Angew. Chem. Int. Ed. Engl.* **48**, 8990–8994 (2009).

32. D. Tanaka, A. Henke, K. Albrecht, M. Moeller, K. Nakagawa, S. Kitagawa, J. Groll, Rapid preparation of flexible porous coordination polymer nanocrystals with accelerated guest adsorption kinetics. *Nat. Chem.* **2**, 410–416 (2010).

33. U. Bentrup, Combining in situ characterization methods in one set-up: Looking with more eyes into the intricate chemistry of the synthesis and working of heterogeneous catalysts. *Chem. Soc. Rev.* **39**, 4718–4730 (2010).

34. D. Perez-Guaita, K. Kochan, M. Martin, D. W. Andrew, P. Heraud, J. S. Richards, B. R. Wood, Multimodal vibrational imaging of cells. *Vib. Spectrosc.* **91**, 46–58 (2017).

35. J. J. Leung, J. Warnan, K. H. Ly, N. Heidary, D. H. Nam, M. F. Kuehnel, E. Reisner, Solar-driven reduction of aqueous CO_2 with a cobalt bis(terpyridine)-based photocathode. *Nat. Catal.* **2**, 354–365 (2019).

36. T. Dou, Z. Li, J. Zhang, A. Evilevitch, D. Kurouski, Nanoscale structural characterization of individual viral particles using atomic force microscopy infrared spectroscopy (AFM-IR) and tip-enhanced raman spectroscopy (TERS). *Anal. Chem.* **92**, 11297–11304 (2020).

37. M. Roman, T. P. Wrobel, C. Paluszkiewicz, W. M. Kwiatek, Comparison between high definition FT-IR, Raman and AFM-IR for subcellular chemical imaging of cholesteryl esters in prostate cancer cells. *J. Biophotonics* **13**, e201960094 (2020).

38. A. Spadea, J. Denbigh, M. J. Lawrence, M. Kansiz, P. Gardner, Analysis of fixed and live single cells using optical photothermal infrared with concomitant Raman spectroscopy. *Anal. Chem.* **93**, 3938–3950 (2021).

39. X. Chang, S. Vijay, Y. Zhao, N. J. Oliveira, K. Chan, B. Xu, Understanding the complementarities of surface-enhanced infrared and Raman spectroscopies in CO adsorption and electrochemical reduction. *Nat. Commun.* **13**, 2656 (2022).

40. X. Li, D. Zhang, Y. Bai, W. Wang, J. Liang, J. X. Cheng, Fingerprinting a living cell by Raman integrated mid-infrared photothermal microscopy. *Anal. Chem.* **91**, 10750–10756 (2019).

41. J. Kneipp, H. Kneipp, K. Kneipp, Two-photon vibrational spectroscopy for biosciences based on surface-enhanced hyper-Raman scattering. *Proc. Natl. Acad. Sci. U.S.A.* **103**, 17149–17153 (2006).

42. K. Hashimoto, V. R. Badarla, A. Kawai, T. Ideguchi, Complementary vibrational spectroscopy. *Nat. Commun.* **10**, 4411 (2019).

43. A. M. Hanninen, R. C. Prince, R. Ramos, M. V. Plikus, E. O. Potma, High-resolution infrared imaging of biological samples with third-order sum-frequency generation microscopy. *Biomed. Opt. Express* **9**, 4807–4817 (2018).

44. A. M. Hanninen, R. C. Prince, E. Potma, Triple modal coherent nonlinear imaging with vibrational contrast. *IEEE J. Sel. Top. Quantum Electron.* **25**, 6800411 (2018).

45. R. Boyd, *Nonlinear Optics* (Academic Press, 1992).

46. J. Sule-Suso, N. Forsyth, V. Untereiner, G. Sockalingum, Vibrational spectroscopy in stem cell characterisation: Is there a niche? *Trends Biotechnol.* **32**, 254–262 (2014).

47. A. P. Bailey, G. Koster, C. Guillermier, E. M. Hirst, J. I. MacRae, C. P. Lechene, A. D. Postle, A. P. Gould, Antioxidant role for lipid droplets in a stem cell niche of Drosophila. *Cell* **163**, 340–353 (2015).

48. A. Pouikli, P. Tessarz, Metabolism and chromatin: A dynamic duo that regulates development and ageing: Elucidating the metabolism-chromatin axis in bone-marrow mesenchymal stem cell fate decisions. *Bioessays* **43**, e2000273 (2021).

49. C. Chonanant, N. Jearanaikoon, C. Leelayuwat, T. Limpaiboon, M. J. Tobin, P. Jearanaikoon, P. Heraud, Characterisation of chondrogenic differentiation of human mesenchymal stem cells using synchrotron FTIR microspectroscopy. *Analyst* **136**, 2542–2551 (2011).

50. S. Brezillon, V. Untereiner, L. Lovergne, I. Tadeo, R. Noguera, F. X. Maquart, Y. Wegrowski, G. D. Sockalingum, Glycosaminoglycan profiling in different cell types using infrared spectroscopy and imaging. *Anal. Bioanal. Chem.* **406**, 5795–5803 (2014).

51. T. Kaletta, M. O. Hengartner, Finding function in novel targets: C. elegans as a model organism. *Nat. Rev. Drug Discov.* **5**, 387–399 (2006).

52. C. W. Freudiger, W. Min, G. R. Holtom, B. Xu, M. Dantus, X. S. Xie, Highly specific label-free molecular imaging with spectrally tailored excitation stimulated Raman scattering (STE-SRS) microscopy. *Nat. Photonics* **5**, 103–109 (2011).

53. C. S. Liao, M. N. Slipchenko, P. Wang, J. Li, S. Y. Lee, R. A. Oglesbee, J. X. Cheng, Microsecond scale vibrational spectroscopic imaging by multiplex stimulated Raman scattering microscopy. *Light Sci. Appl.* **4**, e265 (2015).

54. C. S. Liao, P. Wang, P. Wang, J. Li, H. J. Lee, G. Eakins, J. X. Cheng, Spectrometer-free vibrational imaging by retrieving stimulated Raman signal from highly scattered photons. *Sci. Adv.* **1**, e1500738 (2015).

55. H. Wang, D. Lee, Y. Cao, X. Bi, J. Du, K. Miao, L. Wei, Bond-selective fluorescence imaging with single-molecule sensitivity. *Nat. Photonics* **17**, 846–855 (2023).

56. R. Chikkaraddy, R. Arul, L. A. Jakob, J. J. Baumberg, Single-molecule mid-infrared spectroscopy and detection through vibrationally assisted luminescence. *Nat. Photonics* **17**, 865–871 (2023).

57. J. Yin, L. Lan, Y. Zhang, H. Ni, Y. Tan, M. Zhang, Y. Bai, J. X. Cheng, Nanosecond-resolution photothermal dynamic imaging via MHZ digitization and match filtering. *Nat. Commun.* **12**, 7097 (2021).

58. B. Figueroa, R. Hu, S. G. Rayner, Y. Zheng, D. Fu, Real-time microscale temperature imaging by stimulated Raman scattering. *J. Phys. Chem. Lett.* **11**, 7083–7089 (2020).

59. D. Fu, G. Holtom, C. Freudiger, X. Zhang, X. S. Xie, Hyperspectral imaging with stimulated Raman scattering by chirped femtosecond lasers. *J. Phys. Chem. B* **117**, 4634–4640 (2013).

60. C. Zong, R. Cheng, F. Chen, P. Lin, M. Zhang, Z. Chen, C. Li, C. Yang, J.-X. Cheng, Wide-field surface-enhanced coherent anti-Stokes Raman scattering microscopy. *ACS Photonics* **9**, 1042–1049 (2022).

61. J. Yin, M. Zhang, Y. Tan, Z. Guo, H. He, L. Lan, J. X. Cheng, Video-rate mid-infrared photothermal imaging by single-pulse photothermal detection per pixel. *Sci. Adv.* **9**, eadg8814 (2023).

62. M. N. Slipchenko, R. A. Oglesbee, D. Zhang, W. Wu, J. X. Cheng, Heterodyne detected nonlinear optical imaging in a lock-in free manner. *J. Biophotonics* **5**, 801–807 (2012).

63. C. W. Freudiger, W. Yang, G. R. Holtom, N. Peyghambarian, X. S. Xie, K. Q. Kieu, Stimulated Raman scattering microscopy with a robust fibre laser source. *Nat. Photonics* **8**, 153–159 (2014).

64. I. M. Pavlovetc, E. A. Podshivaylov, R. Chatterjee, G. V. Hartland, P. A. Frantsuzov, M. Kuno, Infrared photothermal heterodyne imaging: Contrast mechanism and detection limits. *J. Appl. Phys.* **127**, 165101 (2020).

65. C. A. Casacio, L. S. Madsen, A. Terrasson, M. Waleed, K. Barnscheidt, B. Hage, M. A. Taylor, W. P. Bowen, Quantum-enhanced nonlinear microscopy. *Nature* **594**, 201–206 (2021).

66. Y. Bi, C. Yang, Y. Chen, S. Yan, G. Yang, Y. Wu, G. Zhang, P. Wang, Near-resonance enhanced label-free stimulated Raman scattering microscopy with spatial resolution near 130 nm. *Light Sci. Appl.* **7**, 81 (2018).

67. P. Fu, W. Cao, T. Chen, X. Huang, T. Le, S. Zhu, D.-W. Wang, H. J. Lee, D. Zhang, Super-resolution imaging of non-fluorescent molecules by photothermal relaxation localization microscopy. *Nat. Photonics* **17**, 330–337 (2023).

68. H. Jang, Y. Li, A. A. Fung, P. Bagheri, K. Hoang, D. Skowronska-Krawczyk, X. Chen, J. Y. Wu, B. Bintu, L. Shi, Super-resolution SRS microscopy with A-PoD. *Nat. Methods* **20**, 448–458 (2023).

69. C. Zong, R. Premasiri, H. Lin, Y. Huang, C. Zhang, C. Yang, B. Ren, L. D. Ziegler, J. X. Cheng, Plasmon-enhanced stimulated Raman scattering microscopy with single-molecule detection sensitivity. *Nat. Commun.* **10**, 5318 (2019).

70. E. Oksenberg, I. Shlesinger, G. Tek, A. F. Koenderink, E. C. Garnett, Complementary surface-enhanced Raman scattering (SERS) and IR absorption spectroscopy (SEIRAS) with nanorods-on-a-mirror. *Adv. Funct. Mater.* **33**, 2211154 (2022).

71. C. Lima, S. Ahmed, Y. Xu, H. Muhamadali, C. Parry, R. J. McGalliard, E. D. Carrol, R. Goodacre, Simultaneous Raman and infrared spectroscopy: A novel combination for studying bacterial infections at the single cell level. *Chem. Sci.* **13**, 8171–8179 (2022).
